# An XDR Pseudomonas aeruginosa ST463 Strain with an IncP-2 Plasmid Containing a Novel Transposon Tn*6485f* Encoding *bla*_IMP-45_ and *bla*_AFM-1_ and a Second Plasmid with Two Copies of *bla*_KPC-2_

**DOI:** 10.1128/spectrum.04462-22

**Published:** 2023-01-18

**Authors:** Yinfei Fang, Nanfei Wang, Zhaoxia Wu, Yijun Zhu, Yongjun Ma, Yue Li, Heng Cai, Piaopiao Zhang, Sebastian Leptihn, Yunsong Yu, Xiaoting Hua, Yuexing Tu

**Affiliations:** a Department of Clinical Laboratory, Affiliated Jinhua Hospital, Zhejiang University School of Medicine, Jinhua, China; b Department of Infectious Diseases, Sir Run Run Shaw Hospital, Zhejiang University School of Medicine, Hangzhou, China; c Key Laboratory of Microbial Technology and Bioinformatics of Zhejiang Province, Hangzhou, China; d Regional Medical Center for National Institute of Respiratory Diseases, Sir Run Run Shaw Hospital, Zhejiang University School of Medicine, Hangzhou, China; e Zhejiang University-University of Edinburgh Institute, Zhejiang University, Haining, China; f University of Edinburgh Medical School, Biomedical Sciences, College of Medicine and Veterinary Medicine, The University of Edinburgh, Edinburgh, United Kingdom; g Department of Critical care medicine, Tongde Hospital of Zhejiang Province, Hangzhou, Zhejiang Province, China; University of Guelph

**Keywords:** *Pseudomonas aeruginosa*, ST463, IncP-2 plasmid, metallo-β-lactamase, KPC-2

## Abstract

The increased carbapenem resistance among Pseudomonas aeruginosa has become a serious health issue worldwide. We reported an extensively drug-resistant (XDR) P. aeruginosa PA30 isolate which belonged to sequence type ST463 and contained an IncP-2 plasmid (pPA30_1) carrying two genes, namely, *bla*_IMP-45_ and *bla*_AFM-1_, which encoded the metallo-β-lactamases AFM-1 and IMP-45, respectively. Additionally, the strain had a plasmid (pPA30_2) with two copies of the *bla*_KPC-2_ genes embedded. The plasmid pPA30_1 was highly similar to the previously reported plasmid pHS17-127, which has the same genetic architecture. This plasmid contained *bla*_IMP-45_, located in a second gene cassette of the integron In*786*, carried by a Tn*1403*-derivative transposon acquiring an IS*CR27n3*-*bla*_AFM-1_ structure. Interestingly, the transposon in pPA30_1 acquired an extra IS*CR1*-*qnrVC6* module and formed a novel transposon, which was subsequently annotated as Tn*6485f*. The *bla*_KPC-2_ genes in pPA30_2 underwent duplication due to the inversion of the IS*26*-*bla*_KPC-2_-IS*26* element, which resulted in two copies of *bla*_KPC-2_.

**IMPORTANCE** The ST463 clone is an emerging high-risk sequence type that is spreading with *bla*_KPC-2_-containing plasmids. The core *bla*_KPC-2_ genetic platform is IS*Kpn27*-b*la*_KPC-2_-IS*Kpn6* in almost all samples, and the adjacent region beyond the core platform varies by IS*26*-mediated inversion or duplication events, amplifying the *bla*_KPC-2_ gene copies. The ST463 P. aeruginosa strain PA30 in our study contains another two metallo-β-lactamase genes, namely, *bla*_IMP-45_ and *bla*_AFM-1_, in a novel transposon Tn*6485f* that is harbored by the IncP-2 megaplasmid. The pPA30_1 carrying *bla*_IMP-45_ and *bla*_AFM-1_ is highly related to pHS17-127 from the ST369 P. aeruginosa strain, indicating the putative dissemination of the megaplasmid between different clones.

## INTRODUCTION

Pseudomonas aeruginosa is an opportunistic pathogen that is one of the most important causes of nosocomial infections. It is a difficult pathogen to eradicate, due to its multidrug resistance to various antimicrobial agents, even to carbapenems (1). The mechanisms of carbapenem resistance mainly include chromosomal mutation-derived membrane permeability alterations that result from porin loss or efflux pump overexpression together with chromosomal cephalosporinase derepression and the production of carbapenemases (2). Metallo-β-lactamases (MBLs), which belong to class B β-lactamases, can hydrolyze all β-lactams, with the exception of aztreonam (3). The Verona integron-encoded metallo-β-lactamase (VIM) types are the most prevalent MBLs produced by P. aeruginosa clinical isolates, and these are followed by the imipenemases (IMPs) (2). Many novel types of MBLs have been reported in China, such as Alcaligenes faecalis MBLs (AFMs) (4). Horizontal gene transfer (HGT) mediated by mobile genetic elements (MGEs), integrons, and transposons, in particular, as well as transmissible resistance plasmids, plays a significant role in the dissemination of carbapenemase genes (5).

Most of the detected transferable plasmids in P. aeruginosa are part of the IncP-2 incompatibility group, which are not readily transferable to E. coli from P. aeruginosa and thus are considered to have a narrow host range (6, 7). Recently, X. Zhang et al. reported a novel subclass B1 MBL AFM-1 (GenBank accession no. AYV97588.1) that is encoded by a *bla*_IMP-45_-containing IncP-2 plasmid, namely, pHS17-127 (GenBank accession no. CP061377), from a clinical carbapenem-resistant P. aeruginosa (CRPA) (8). Here, we report an extensively drug-resistant (XDR) P. aeruginosa strain, which we called PA30 in a molecular epidemiologic study, that belonged to the novel, highly epidemic sequence type ST463. In this work, we analyze the complete genetic makeup of the XDR P. aeruginosa strain PA30, and we also provide a detailed genetic characterization of two plasmids.

## RESULTS

### Clinical and microbiological characteristics of the P. aeruginosa isolate PA30.

The PA30 strain was isolated from a catheter reserved in a 67-year-old male patient who suffered from an operation of clearance of intracranial hematoma after being admitted to a hospital in Jinhua, Zhejiang. The patient had a fever of 37.8°C after the operation, and he was successively treated with cephalosporins, linezolid, and meropenem before the urine sample was collected during the hospitalization. Combined with the clinical manifestations of the patient, the isolated P. aeruginosa was related more to colonization than to infection. Eventually, the patient recovered after the therapy.

Antimicrobial susceptibility tests of PA30 exhibited an XDR phenotype (9). It demonstrated resistance to piperacillin, cefepime, ceftazidime, piperacillin-tazobactam, imipenem, meropenem, aztreonam, amikacin, gentamicin, levofloxacin, ciprofloxacin, and even the most novel β-lactam/β-lactamase inhibitor combinations (ceftazidime-avibactam, imipenem/relebactam, meropenem-vaborbactam, and ceftolozane-tazobactam), and it only remained susceptible to colistin (Table S1).

The sequence type of the PA30 isolate was identified as ST463, which was first reported in 2015 in China and has become a potential regional high-risk clone of P. aeruginosa (10, 11). Our whole-genome sequencing analysis of the strain PA30 revealed a 6.92 Mb chromosome and two plasmids, which we called pPA30_1 and pPA30_2. We identified the *bla*_IMP-45_ and *bla*_AFM-1_ genes on pPA30_1 and two copies of the *bla*_KPC-2_ gene on pPA30_2. Abundant virulence genes were detected in PA30, especially the genes *exoU* and *exoS* that encode the type III secretion system effectors, which are considered to be the attribution of the hypervirulent phenotype of ST463 P. aeruginosa.

In more than three independent conjugation experiments, neither of the transconjugants of these two plasmids grew on the selective plates. The growth curves demonstrated the impaired growth of PA30 (Fig. S1A), consistent with the decreased growth rate compared to ZYPA30 (*P = *0.0024) (Fig. S1B).

### Features of plasmid pPA30_1.

**FIG 1:**
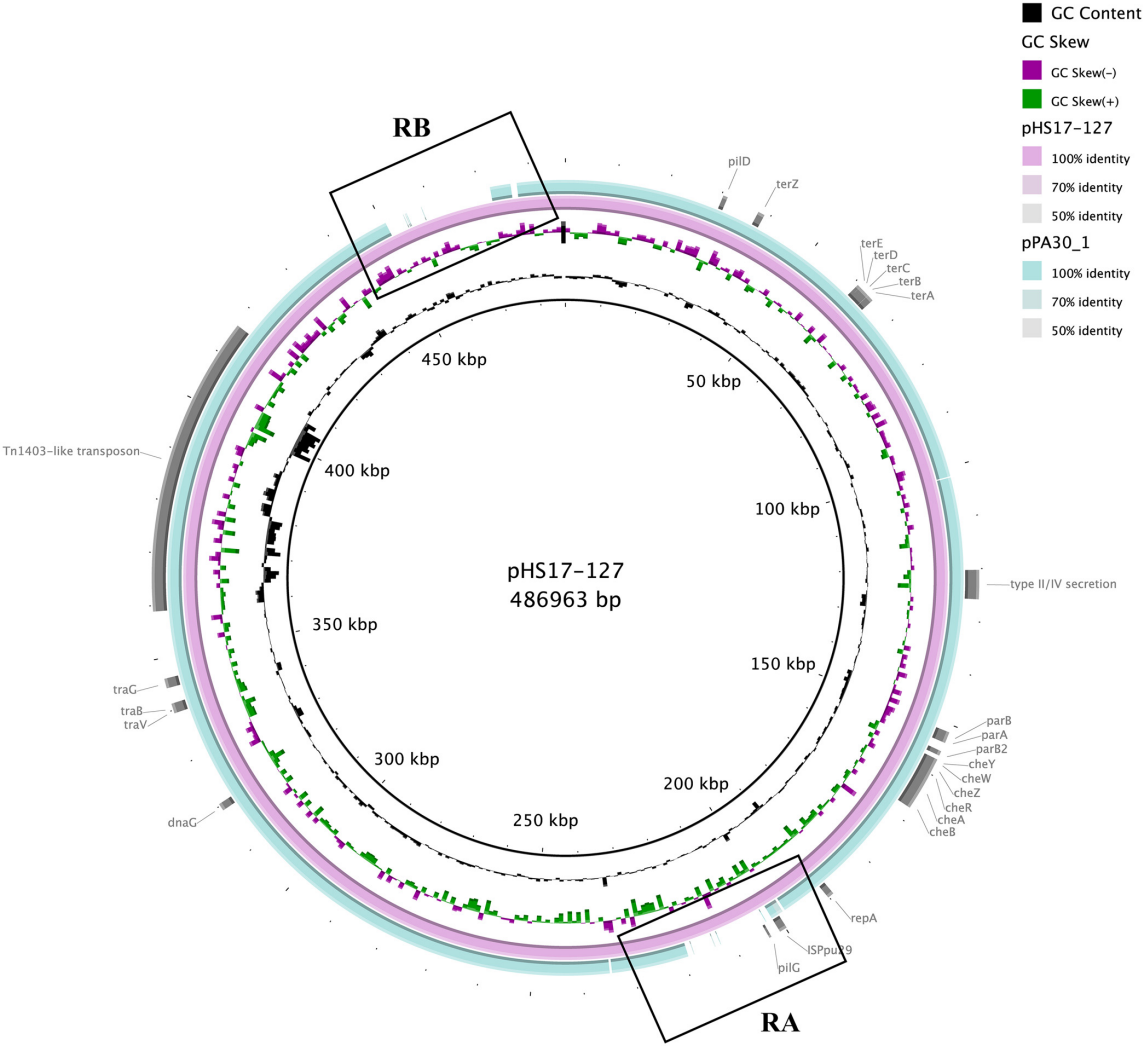
Genetic organization of the pPA30_1 plasmid and comparison with the pHS17_127 plasmid. The sequence of pHS17_127 is taken as the reference, and the innermost circle indicates the scale. The second and third circles illustrate the GC content deviation from the average in the reference genomes and the GC skew, respectively. The colored circles from the inner to the outer represent each plasmid, as shown in the right column. The annotations on the outermost circle indicate the locations of the main features of pHS17_127. The solid regions demonstrate a sequence similar to that of pHS17_127, whereas the gaps represent regions lacking sequence similarity. Region A and Region B are marked by two boxes named RA and RB.

Plasmid pPA30_1 had a length of 453,250 bp and a guanine-cytosine (GC) content of 56%. According to BLASTn results, the plasmid pPA30_1 was highly similar to the aforementioned plasmid pHS17-127 (99% query cover and 100% nucleotide similarity) (Fig. 1). These two plasmids encoded identical RepA replication proteins as well as identical ParA and ParB partition proteins (100% amino acid sequence identities), suggesting that pPA30_1 also belonged to the IncP-2 group. Both plasmid backbones contained genes that are typically found in IncP-2 type plasmids. Besides the aforementioned replication (*repA*) and partitioning (*parA* and *parB*) genes, which are responsible for the replication region and the partition system, respectively, conjugative transfer region genes (*traGBV*, *dnaG*, and type IV pilus-related/type II secretion genes) that enable horizontal transmission to other bacterial cells were also identified in pPA30_1. The chemotaxis operon (*che*BARZWY), which encodes a type IV pilus (T4aP) to modulate twitching motility via a mechanism of extension-retraction, was found. The heavy metal resistance operon *ter*ABCDEZ, encoding tellurite resistance, was also identified (Fig. 1). A direct comparison between pHS17-127 and pPA30_1 revealed that two regions were absent from pPA30_1, marked as region A (subsequently referred to as RA, 19 kbp) and region B (subsequently referred to as RB, 21 kbp) in Fig. 1.

**FIG 2:**
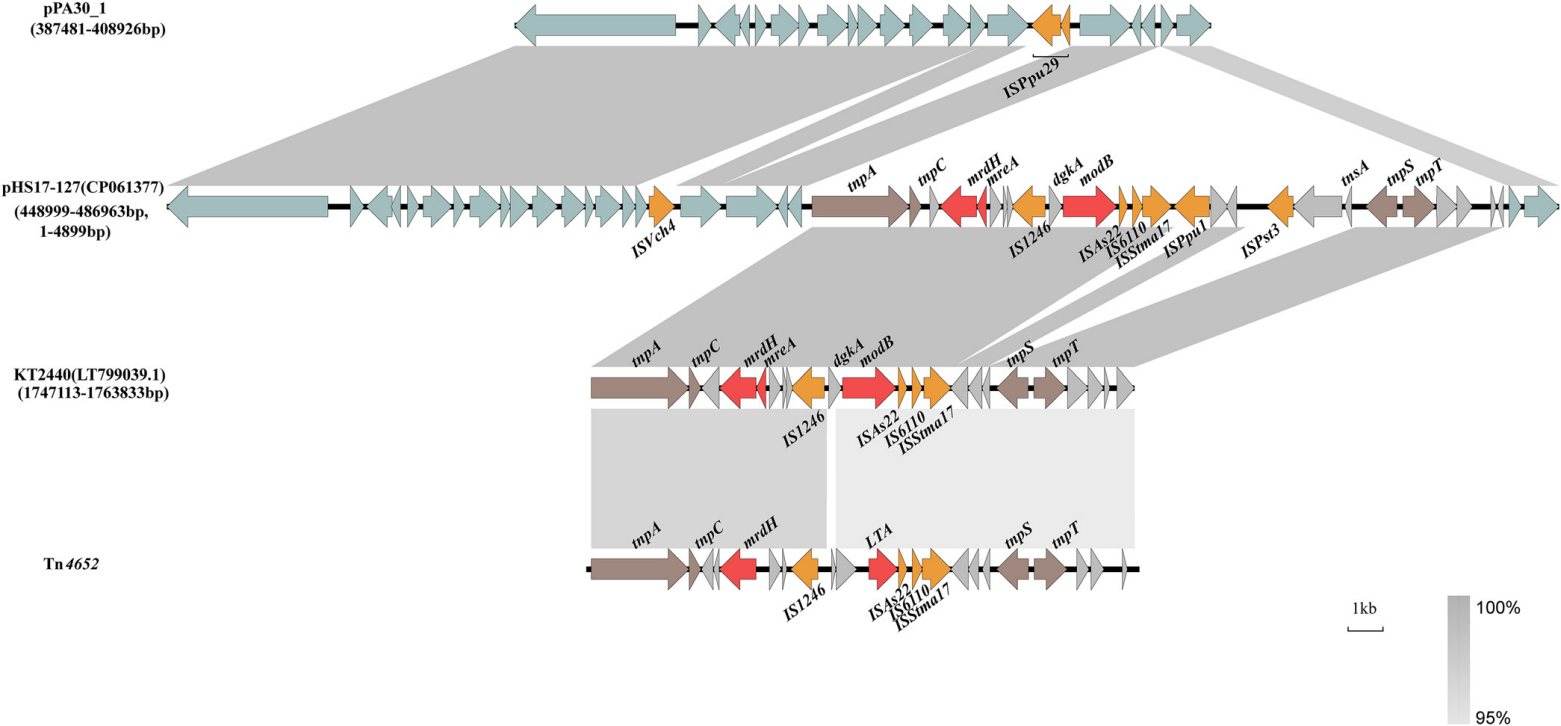
Alignment of the genetic context of RB and the related sequence from KT2400. Transposon Tn*4652* is used as a reference. Heavy metal resistance genes are denoted by red arrows. Transposons and IS elements are denoted by brown and orange arrows. The light blue arrows indicate the identical backbones of pPA30_1 and pHS17-127.

7 plasmids were found to contain regions with a high similarity to RA, displaying 93% coverage and 100% nucleotide identity. All, save one, were found in P. aeruginosa strains, whereas the exception was isolated from Pseudomonas putida. The *pil* operon *pilD* was found in RA, and no known antimicrobial resistance genes were identified in this region. BLASTx results showed that the RA region lacking in pPA30_1 was flanked by the IS*66*-like element IS*Ppu30*, which was disrupted by IS*Ppu29* (IS*3* family member). With 84% query coverage and 99.99% nucleotide similarity, RB was highly related to the chromosome of the P. putida strain KT2440 (GenBank accession number: LT799039.1), a plasmid-free derivative of a toluene-degrading bacterium (12). Structurally, the RB module was similar to the transposon Tn*4652*, part of the Tn*3* family of transposons (Fig. 2) (13). Tn*3*-family transposons typically include a *tnpA* transposase gene that catalyzes the generation of a cointegrate structure, a *tnpR* resolvase gene, and a resolution (*res*) site that resolves the cointegrate into separate molecules (7). However, Tn*4652*, as a deletion derivative of Tn*4651* deleting all toluene-catabolic genes, was composed of two genes, namely, *tnpA*, and *tnpC*, in the step of cointegrate-formation, as well as two other genes, namely, *tnpS*, and *tnpT*, for the cointegrate-resolution process (14, 15). This genetic organization mediated part of the transposition of Tn*4652*. The heavy metal resistance gene *mrdH*, which is responsible for nickel, cadmium, and zinc resistance, was identified in this transposon. Next to the gene, we found *mreA*, which encodes a metal resistance-associated cytoplasmic protein. As this transposon structure was absent in pPA30_1, the plasmid might not confer resistance to nickel, cadmium, and zinc, in contrast to pHS17-127.

### Antibiotic resistance region of plasmid pPA30_1.

**FIG 3:**
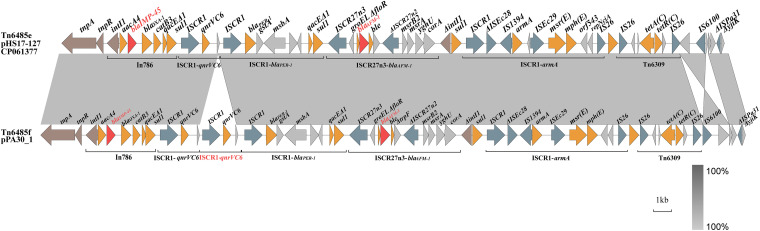
Alignment of the genetic context of Tn*6485e* from the pHS17-127 and Tn*6485f* of the plasmid pPA30_1. Shaded regions denote nucleotide identity (100%). Red arrows denote the genes *bla*_IMP-45_ and *bla*_AFM-1_. The other antibiotic resistance genes are denoted by orange arrows. Brown arrows denote mobile elements. IS elements are highlighted in blue.

A 60,801 bp long region containing multidrug resistance determinants was detected in pPA30_1. The element was identified as a Tn*1403*-like transposon that was frequently found in IncP-2-derivative plasmids encoding *bla*_IMP-45_ in Pseudomonas species. It exhibited similarity to Tn*6485e* described in pHS17-127, displaying 99% coverage and 99.99% nucleotide identity. The *bla*_IMP-45_ gene was located within In*786*, a class 1 integron containing 5 antimicrobial resistance cassettes arranged one after the other. The genes *aacA4*-*bla*_IMP-45_-*gcu3*-*bla*_OXA-1_-*catB3* conferred resistance to aminoglycosides, carbapenems, and chloramphenicol. Upstream of the cassette array were *intI1* flanked by a Tn*1403*-like transposon containing *tnpAR* and the 3′CS, including the *qacEΔ1* gene, and this was followed by *sul1*, which encodes resistance to early sulfonamide antibiotics (Fig. 3).

The genetic architecture of *bla*_AFM-1_ in pPA30_1 was identical to that in pHS17-127, which was identified as a conserved region of *groEL/*Δ*groEL-*Δ*floR-bla*_AFM-1_*-ble-*Δ*trpF-*ΔIS*CR27n2-*ΔIS*Pme1-msrB2-msrA-yghU-corA*, bracketed by two IS*CR27*-like elements (IS*CR27n3* and ΔIS*CR27n1*). Compared with Tn*6485e*, which has been described in detail, regarding the evolution of the element (8), the novel transposon Tn*6485f* detected in pPA30_1 was related to the IS*CR1*-*qnrVC6* and the IS*CR1*-*bla*_PER-1_ modules, particularly the region between In*786* and the IS*CR27n3*-b*la*_AFM-1_ module. Two tandem repeats of IS*CR1*-*qnrVC6* modules were observed upstream of the IS*CR1*-*bla*_PER-1_ module in Tn*6485f*, which might have been generated by the IS*CR1* elements’ transferability of adjacent DNA sequences (7). Downstream from the IS*CR27n3*-b*la*_AFM-1_ module in both Tn*6458e* and Tn*6458f*, the genetic platforms were almost identical, being composed of the IS*CR1*-associated *armA* module (IS*CR1*-ΔIS*Ec28*-IS*1394*-*armA*-IS*Ec29*-*msr(E)*-*mph(E)*-*orf543*-*repNciA*-IS*26*) that was followed by an IS*26*-composite transposon Tn*6179* (IS*26*-*aph(3′)-Ia*-IS*26*) and an IS*26*-composite transposon Tn*6309b* carrying the tetracycline resistance module *tetAR(C)* (Fig. 3).

### Features of the pPA30_2 plasmid.

**FIG 4:**
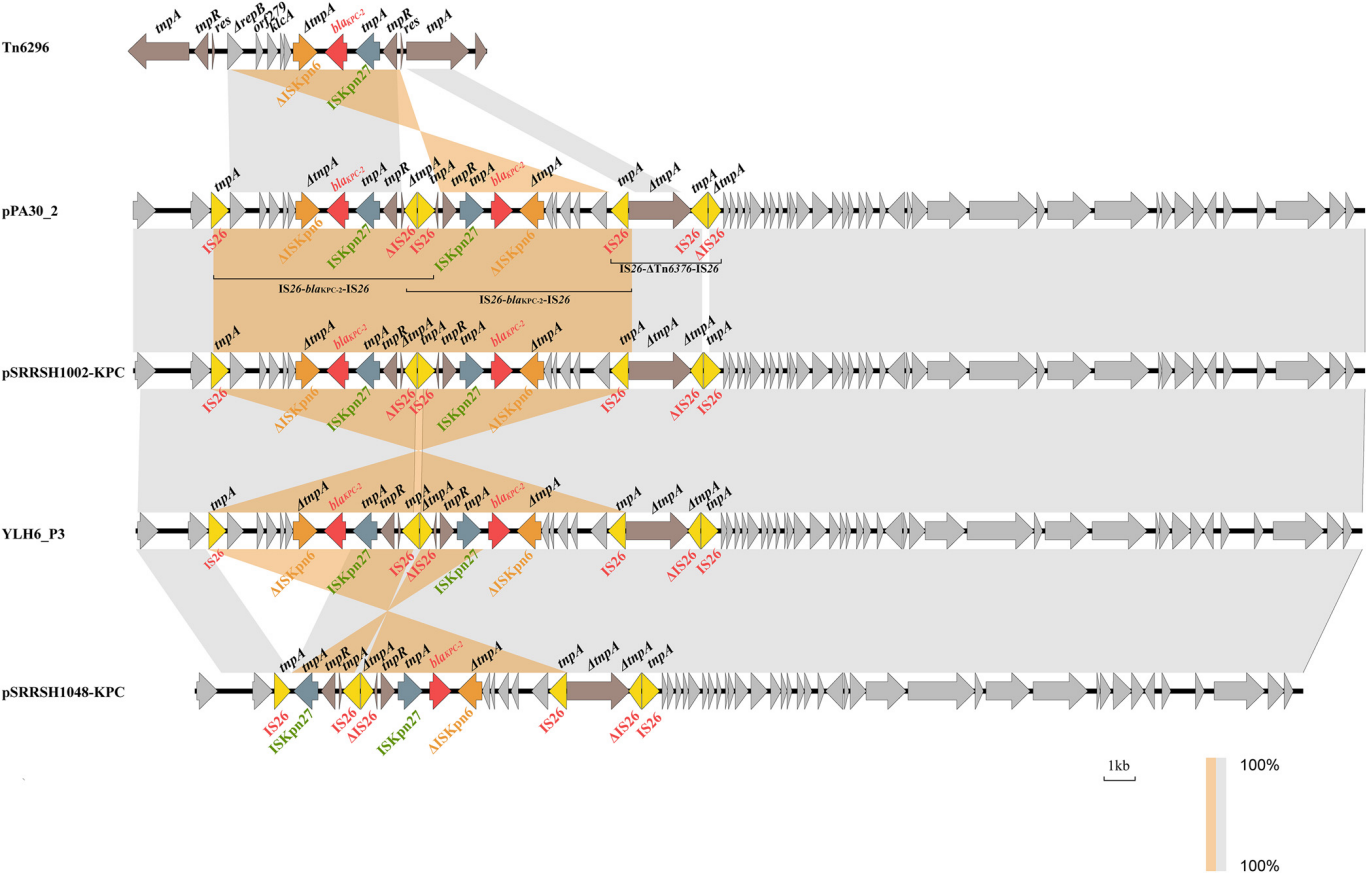
Alignment of the genetic context of pPA30_2 and highly similar plasmids. Transposon Tn*6292* is used as a reference. The antibiotic resistance genes *bla*_KPC-2_ are denoted by red arrows. The IS elements IS*26*, IS*Kpn27*, and ΔIS*Kpn6* are denoted by yellow, blue, and orange arrows, respectively.

The complete plasmid sequence of pPA30_2 was 49,370 bp long, with an average GC content of 58%. A BLAST analysis revealed that pPA30_2 was identical to pSRRSH1002-KPC, YLH6_P3, and pSRRSH1048-KPC (100% nucleotide identity and query coverage, accession numbers: CP064398.1, MK882885.1, and CP064396.1, respectively), all of which were isolated from P. aeruginosa in Hangzhou, China, and identified as the ST463 sequence type. Separated by an identical backbone, the accessory regions of these four plasmids were composed of two IS*26*-associated modules, namely, the IS*26*-*bla*_KPC-2_-IS*26* and IS*26*-ΔTn*6376*-IS*26*, which originated from Tn*6296* (Fig. 4). The *bla*_KPC-2_ gene in Tn*6296* was flanked by IS*Kpn27* and ΔIS*Kpn6*, and this was followed by the *korC-orf6-klcA-ΔrepB* genes.

The *bla*_KPC-2_-associated genetic environments in pPA30_2 and pSRRSH1002-KPC were identical to those of Tn*6296*. The bordering sequences of the elements indicated the insertion of two IS*26* elements forming the IS*26*-*bla*_KPC-2_-IS*26* unit. This was accompanied by the duplication and the inversion of this unit, mediated by IS*26*, which generated two symmetric structures, resulting in two copies of the *bla*_KPC-2_ genes. The adjacent IS*26* regions of two IS*26*-*bla*_KPC-2_-IS*26* units overlapped each other, resulting in mosaic structures. In YLH6_P3, IS*26*-mediated inversion events caused a horizontal flip of the platform flanked by IS*26* elements carrying two copies of *bla*_KPC-2_. Compared with YLH6_P3, the plasmid pSRRSH1048-KPC lost a *bla*_KPC-2_ gene in an IS*26*-*bla*_KPC-2_-IS*26* unit, which likely occurred due to an IS*26*-mediated inversion event.

## DISCUSSION

The XDR P. aeruginosa PA30 was resistant to all of the β-lactams and the β-lactam/β-lactamase inhibitor combinations that were tested, including ceftazidime-avibactam (CAZ-AVI). The overexpression of the *bla*_KPC-2_ gene has been implicated as a potential resistance mechanism to CAZ-AVI, particularly when present in multiple gene copies (14, 16). Modifications in membrane permeability, including the overexpression of efflux pumps and the decreased expression and/or mutations in porin genes, are another mechanism that seems to be related to the increase of the CAZ-AVI minimum inhibitory concentration (MIC) (14). In our study, because of the existence of the MBLs, it is reasonable for PA30 to have a high MIC for CAZ-AVI. The CAZ-AVI resistance conferred by the AFM-1 cloning strains has been demonstrated in previous study (8).

The ST463 clone is an emerging high-risk sequence type that is spreading with *bla*_KPC-2_-containing plasmids (15). The surveillance of KPC-producing P. aeruginosa (KPC-PA) isolates from several hospitals in China demonstrated that ST463 was the dominant CRPA clone in East China, and it accounts for 70.9% of 151 KPC-PA strains (16). The major plasmid type carrying *bla*_KPC-2_ that is found in the ST463 strains is the type I plasmid. The core *bla*_KPC-2_ genetic platform is IS*Kpn27*-b*la*_KPC-2_-IS*Kpn6* in almost all samples, and the adjacent region beyond the core platform varies by IS*26*-mediated inversion or duplication events, which amplify the *bla*_KPC-2_ gene copies (16). These findings are consistent with the event in our study. IS*26*-dominated mobile elements appear to promote *bla*_KPC-2_ transmission in ST463. Conversely, the clone ST463 exhibiting extensive drug resistance contributes to its survival in hospital environments and facilitates the spread of the *bla*_KPC-2_ gene in P. aeruginosa.

However, the spread and evolution of bacterial resistance are always more complex than are imagined. The *in vivo* evolution driving the acquisition of *bla*_KPC-2_ under carbapenem exposure in a *bla*_AFM-1_-harboring ST463 P. aeruginosa strain has been reported (17). The ST463 P. aeruginosa strain in our study contains two MBLs genes, namely, *bla*_IMP-45_ and *bla*_AFM-1_, and these are harbored by the IncP-2 megaplasmid. The impaired growth rate of PA30 indicates that the acquisition of *bla*_IMP-45_ and *bla*_AFM-1_ may impose fitness costs upon the host bacteria. The IncP-2 megaplasmids share a highly similar core genetic backbone, but they are flexible in the AMR gene regions. In general, the plasmids capture the resistance genes with the help of mobile elements, such as the transposon Tn*6485f* and the IS*CR27n3*-*bla*_AFM-1_ module in our study, and they act as vehicles disseminated within and among species (18). The transmission of the genes encoding MBLs related to the spread of IncP-2 megaplasmids has been reported in recent years (19, 20). The pPA30_1 carrying *bla*_IMP-45_ and *bla*_AFM-1_ is highly related to pHS17-127 from the ST369 P. aeruginosa strain, indicating the putative dissemination of the megaplasmid between different clones. It is predicted that the HGT of megaplasmids occurs mainly via conjugation, with experiments showing conjugation at high rates (21). However, some megaplasmids appear to lack conjugative machinery and achieve conjugation with the help of other self-transmissible plasmids (22). Although the conjugative transfer region genes are identified in pPA30_1, the genome sequence similarity and GC content similarity can be strong barriers to HGT in prokaryotes (23). The transmission of plasmids may be more complicated in a clinical setting, as various evolutionary pressures should be taken into consideration.

In conclusion, we report a clinical, extensively drug-resistant (XDR) P. aeruginosa belonging to sequence type ST463 that contains two plasmids. One plasmid is an IncP-2 megaplasmid containing a *bla*_IMP-45_-harboring In*786* integron and a *bla*_AFM-1_ gene embedded in an IS*CR27*-like structure. The second plasmid is associated with an IS*26*-mediated gene duplication that results in two copies of *bla*_KPC-2_. We investigated the putative megaplasmid-associated spread of MBL genes in ST463 P. aeruginosa. The occurrence of such strains illustrates the urgent need to continue epidemiologic studies in order to understand the spread of these multiresistant P. aeruginosa ST463 strains.

## MATERIALS AND METHODS

### Bacterial strain and antibacterial susceptibility determination.

The clinical P. aeruginosa PA30 was identified in a molecular epidemiologic study in 2021. The MICs of antibiotics against strain PA30 were determined via the agar dilution method, and the results were interpreted according to the breakpoints recommended by the 2022 Clinical and Laboratory Standards Institute (CLSI) guidelines (24). The MICs of imipenem/relebactam, meropenem-vaborbactam, and ceftolozane-tazobactam were determined using a Thermo Scientific Sensititre Susceptibility Plate (Thermo, CHN3SRRS), and the results were interpreted according to the standard reference card.

### Genome sequencing and plasmid analysis.

Total DNA was extracted using the QIAamp DNA Minikit (Qiagen, Hilden, Germany), according to the manufacturer’s instructions, and sequenced using an Illumina HiSeq X10 platform (San Diego, CA, United States). The long-read Oxford Nanopore Technologies MinION platform was used after treatment with a supplementary sequencing kit (Nanopore, Oxford, United Kingdom). Both short and long reads were *de novo* hybrid assembled using Unicycler v0.4.8 (25) and then annotated using RAST 2.0 (26) and Prokka 1.14.6 (27). This was followed by a manual review through BLASTn/BLASTp (28). Multilocus sequence typing (MLST) was performed using the PubMLST (29). Resfinder (30) and ABRicate v0.8.13 (https://github.com/tseemann/abricate) were used to identify resistance genes and virulence genes. Transposon Registry (31), ISfinder (32), and INTEGRALL (33) were utilized for the mobile genetic elements. The circular genome map and comparative genome map were completed using Easyfig and BRIG (34).

### Conjugation experiments.

Conjugation experiments were performed with clinical isolate PA30 as the donor strain and a rifampin-resistant derivative of P. aeruginosa PAO1 as the recipient strain. The selective Mueller-Hinton (MH) agar plates were supplemented with rifampicin (800 μg/mL) and meropenem (2 μg/mL) or meropenem (8 μg/mL). The colonies of donor and recipient bacteria were cultured in 2 mL Luria-Bertani (LB) medium and shaken at 37°C for 4 h. The donor and recipient bacteria in LB broth were combined at a 1:1 ratio (500 μL, respectively) in 4 mL LB broth, and they were then cocultured at 37°C without agitation. After 24 h, the mixture was resuspended and plated onto the selective Mueller-Hinton agar plates and incubated at 37°C overnight. The growing colonies on the selective agar plates were confirmed via polymerase chain reaction (PCR) amplification. Each conjugation experiment was repeated at least three times.

### Growth kinetics and statistics.

A noncompetitive growth kinetics analysis was conducted to evaluate the fitness of the PA30 strain. The reference strain, namely, the ST463 P. aeruginosa ZYPA28 harboring *bla*_KPC-2_ (GenBank assembly accession: GCA_020036495.1), was selected from the strains in our previous surveillance study, based on the smallest single nucleotide polymorphism (SNP) differences (SNP = 2). 3 colonies of each strain were cultured independently in 2 mL LB medium overnight, and they were then diluted to 1:100 in MH broth. A total of 200 μL of each diluted culture was added into a 100-well plate in 3 replicates and shaken at 37°C. A Bioscreen C Analyzer (Oy Growth Curves Ab. Ltd., Finland) was used to record the optical density at 600 nm (OD_600_) of each culture every 5 min for 20 h. The growth rate based on the OD_600_ curves was calculated using an R script. The statistical analysis was performed using GraphPad Prism v9. Unpaired *t* tests were used to evaluate the differences between the means. *P* values of <0.05 were considered to be indicative of a statistically significant result.

### Data availability.

The data in our research are available to access. The complete sequences of the plasmids pPA30_1 and pPA30_2 have been submitted to GenBank under the accession numbers CP104871 and CP104872, respectively. The accession number of the PA30 chromosome is CP102441.
