# Supplementary material for: An XDR Pseudomonas aeruginosa ST463 Strain with an IncP-2 Plasmid Containing a Novel Transposon Tn6485f Encoding blaIMP-45 and blaAFM-1 and a Second Plasmid with Two Copies of blaKPC-2
Source: Microbiol Spectr. 2023 Jan 18;11(1):e04462-22. doi: 10.1128/spectrum.04462-22 (PMC9927494; doi:10.1128/spectrum.04462-22)
Supplement: Supplemental file 1 — Supplemental material. Download spectrum.04462-22-s0001.pdf, PDF file, 0.2 MB [file spectrum.04462-22-s0001.pdf]

Table S1. MICs for Pseudomonas aeruginosa clinical isolate PA30

Antibiotic	**PIP**	**FEP**	**CAZ**	**PTZ**	**CZA**	**IMP**	**MEM**	**AZT**	**AK**	**GM**	**LEV**	**CIP**	**IMR**	**MEV**	**C/T**	**COL**
MICs (mg/L)	>128	>128	>128	>128	>128	>128	>128	>128	>128	>128	>128	16	>128	>64	>128	1

PIP, piperacillin; FEP, cefepime; CAZ, ceftazidime; PTZ, piperacillin-tazobactam; CZA, ceftazidime-avibactam; IMP, imipenem; MEM, meropenem; AZT, aztreonam; AK, amikacin; GM, gentamicin; LEV, levofloxacin; CIP, ciprofloxacin; IMR, imipenem/relebactam; MEV, meropenem/vaborbactam; C/T, ceftolozane/tazobactam；COL, colistin.

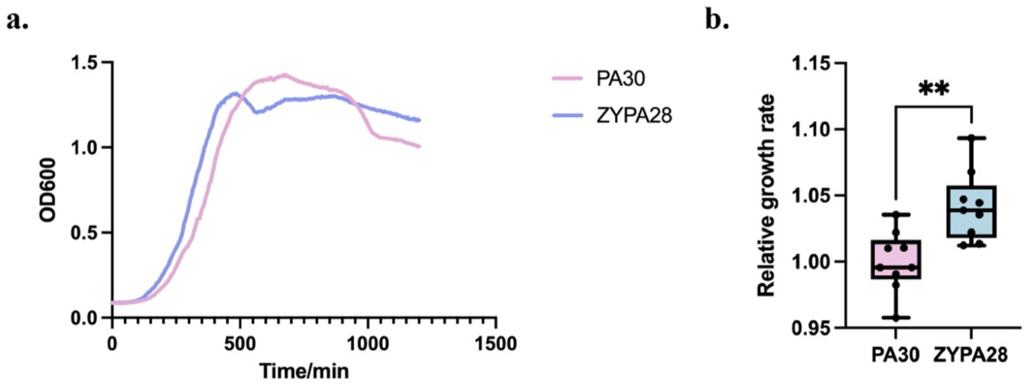

Figure S1. A growth kinetics of PA30 and ZYPA28. (a) Growth curves of PA30 and ZYPA28 in MH broth medium without antibiotics. (b) The growth rate of PA30 is normalized to 1, and the relative growth rates of PA30 and ZYPA28 are expressed as means ± standard deviations. Each point represents a data value.
